# High prevalence of Phasi Charoen-like virus from wild-caught *Aedes aegypti* in Grenada, W.I. as revealed by metagenomic analysis

**DOI:** 10.1371/journal.pone.0227998

**Published:** 2020-01-31

**Authors:** Maria E. Ramos-Nino, Daniel M. Fitzpatrick, Scott Tighe, Korin M. Eckstrom, Lindsey M. Hattaway, Andy N. Hsueh, Diana M. Stone, Julie Dragon, Sonia Cheetham

**Affiliations:** 1 Department of Microbiology, School of Medicine, St. George’s University, St. George's, Grenada, West Indies; 2 Department of Pathobiology, School of Veterinary Medicine, St. George’s University, St. George's, Grenada, West Indies; 3 University of Vermont Massively Parallel Sequencing Facility, Burlington, Vermont, United States of America; Universita degli Studi di Camerino, ITALY

## Abstract

Arboviruses cause diseases of significant global health concerns. Interactions between mosquitoes and their microbiota as well as the important role of this interaction in the mosquito's capacity to harbor and transmit pathogens have emerged as important fields of research. *Aedes aegypti* is one of the most abundant mosquitoes in many geographic locations, a vector capable of transmitting a number of arboviruses such as dengue and Zika. Currently, there are few studies on the metavirome of this mosquito particularly in the Americas. This study analyzes the metavirome of *A*. *aegypti* from Grenada, a Caribbean nation with tropical weather, abundant *A*. *aegypti*, and both endemic and arboviral pathogens transmitted by this mosquito. Between January and December 2018, 1152 mosquitoes were collected from six semi-rural locations near houses in St. George Parish, Grenada, by weekly trapping using BG-Sentinel traps. From these, 300 *A*. *aegypti* were selected for analysis. The metavirome was analyzed using the Illumina HiSeq 1500 for deep sequencing. The generation sequencing library construction protocol used was NuGEN Universal RNA with an average read length of 125 bp. Reads were mapped to the *A*. *aegypti* assembly. Non-mosquito reads were analyzed using the tools FastViromeExplorer. The NCBI total virus, RNA virus, and eukaryotic virus databases were used as references. The metagenomic comparison analysis showed that the most abundant virus-related reads among all databases and assemblies was Phasi Charoen-like virus. The Phasi Charoen-like virus results are in agreement to other studies in America, Asia and Australia. Further studies using wild-caught mosquitoes is needed to assess the impact of this insect-specific virus on the *A*. *aegypti* lifecycle and vector capacity.

## Introduction

The most common mosquito-borne arbovirus diseases are transmitted by two genera of mosquitoes: *Aedes* and *Culex*[1–6]. The public health impact of these arboviruses has increased dramatically over the past years, spreading to new geographic locations and increasing in incidence within their range [7]. *Aedes* mosquitoes together with *Culex quinquefasciatus* are important vector mosquitoes in the Americas, and are the most abundant mosquitoes found in populated areas in Grenada, West Indies [8]. From the genera *Aedes*, two specific species, *Aedes aegypti* and *Aedes albopictus* have been shown to be important vectors of globally important arboviruses including the dengue viruses (DENV) [9], yellow fever virus [10], Zika virus (ZIKV) [11], and chikungunya virus [12].

Preventive treatments against mosquito-borne diseases are mostly limited to personal protection and mosquito population control using insecticides [13]. However, intensive and repeated insecticide use leads to the development of mosquito resistance, which has been reported to have a significant impact on transmission dynamics of microorganisms. For instance, a study on the effects of insecticide resistance mechanisms on vector competence of the mosquito *C*. *quinquefasciatus* demonstrated dissemination of West Nile Virus (WNV) in the mosquito body, leading to an increase in transmission efficiency by resistant mosquitoes [14]. Similarly, current research continues to elucidate interactions among mosquitoes, their endogenous microbiota, and other pathogens they transmit. In *A*. *aegypti*, the microbiota in the mosquito has been reported to influence the susceptibility to infection to arboviruses [13,15,16]. For example, DENV replication has been reported to be affected by gut bacteria [17,18] which exert antiviral activity through mechanisms not completely understood [13,19–21], but that may be indirectly associated to innate antiviral responses and antimicrobial peptides by the gut microbiota [17]. Studies on the microbiome reinforce the great potential for the development of microbial-based strategies to control vector-borne pathogens [13].

In recent years, metagenomics has emerged as a powerful tool to study microbial diversity in a culture independent manner [22], an approach that is helping define the microbiome of mosquitoes [23–26]. Part of the uncovered microbiome is the insect-specific viruses (ISVs), viruses restricted to arthropods that are unable to replicate in vertebrate cells [27]. Insect-specific viruses are highly prevalent in wild mosquito populations [25,28–31] and have been reported to suppress [32–36], enhance [37,38], or have no effect [39] on replication of medically important arboviruses, potentially affecting vector competence [40–42].

There is a potential use of ISVs as biocontrol agents. The close genetic similarity between ISVs and arboviruses have a potential for interference in their replication either through upregulation of antiviral immune responses in the vector or via superinfection exclusion (i.e., homologous interference), where similar viruses can block each other through competition [42,43].

The present study sheds some light on the virome of *A*. *aegypti* mosquito in Grenada, an endemic region for several medically important arboviruses in the Caribbean.

## Material and methods

### Mosquito collection

**Fig 1:**
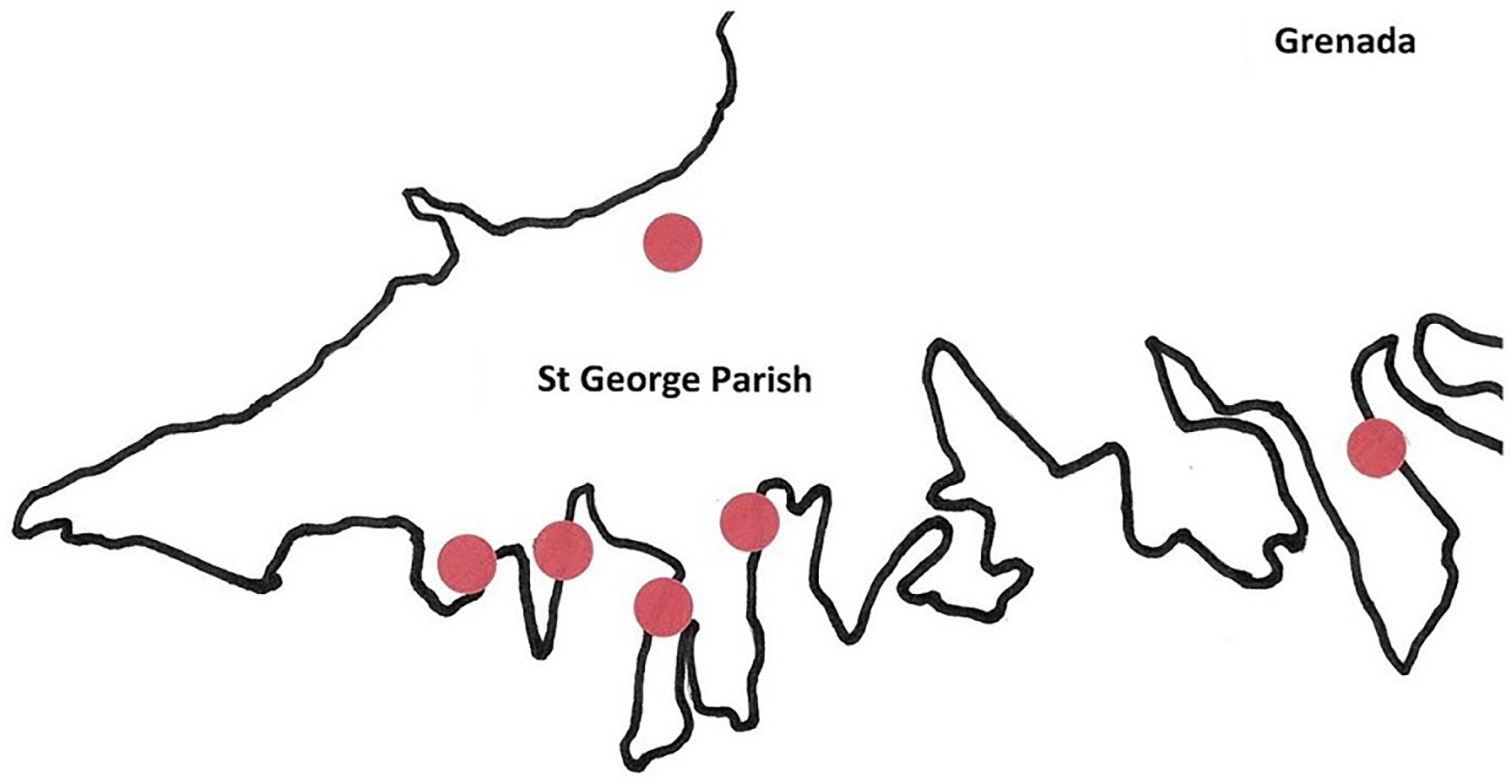
Map of collection sites in St. George Parish, Grenada.

Three hundred female *A*. *aegypti* mosquitoes were randomly selected out of 1,152 mosquitoes collected between January 2018 and December 2018, twice each week from six semi-rural locations in St. George Parish, Grenada (12°15'46'' N 61°36'15'' W) (Fig 1). Biogents Sentinel (Biogents, Regensburg, Germany) traps baited with octenol and yeast-based carbon dioxide attractants [44] were placed within 3 m of houses to attract mosquitoes. After 24 h, traps were collected, and mosquitoes were stored at −80°C. Subsequently, mosquitoes were identified to species by morphological analysis. Because morphological keys that include recently introduced invasive taxa are not available for Caribbean islands, identification keys in Darsie and Ward [45] and in Rueda [46] were used to discriminate between species known to occur in Grenada based on the Walter Reed Biosystematics Unit [47]. Mosquitoes were then placed in RNAlater® (Sigma Aldrich, St. Louis, Missouri, USA) until further processing. Before RNA extraction, mosquito heads were removed using a sterile scalpel blade to prevent PCR inhibition [48]; wings and legs were also removed to reduce host RNA. No IACUC was required for the use of mosquitoes in this study.

### Total RNA extraction and RNA-Seq

RNA extraction was performed in batches of 30 mosquitoes at a time (ten pools) using TRIzol (ThermoFisher, Carlsbad, California, USA). Invitrogen^™^ Phasemaker^™^ Tubes (ThermoFisher) were used for the phase separation. RNA was DNase-treated using TURBO DNA-*free*^™^ (ThermoFisher) and RNA quality was evaluated utilizing an Agilent 2100 Bioanalyzer (Agilent, Santa Clara, California, USA) as previously described [49].

All samples were pooled for library construction. Library construction was performed using the NuGEN Tecan universal RNA sequencing reagents custom ribosomal depletion probes (AnyDeplete^™^) against *A*. *aegypti* rRNA including 5.8S, 18S, 28S, ITS1, ITS2. The specific accession number used for the depletion probes included: AAEL028668-RA AAEL028668-RA-E1_exon:rRNA; NIGP01001771 dna:scaffold scaffold:AaegL5:NIGP01001771:6654:6806:1; AAEL028730-RA AAEL028730-RA-E1 exon:rRNA; NIGP01001771 dna:scaffold scaffold:AaegL5:NIGP01001771:12876:14043:1; AAEL028771-RA AAEL028771-RA-E1 exon:rRNA; NIGP01001771 dna:scaffold scaffold:AaegL5:NIGP01001771:7002:11112:1.

**Fig 2:**
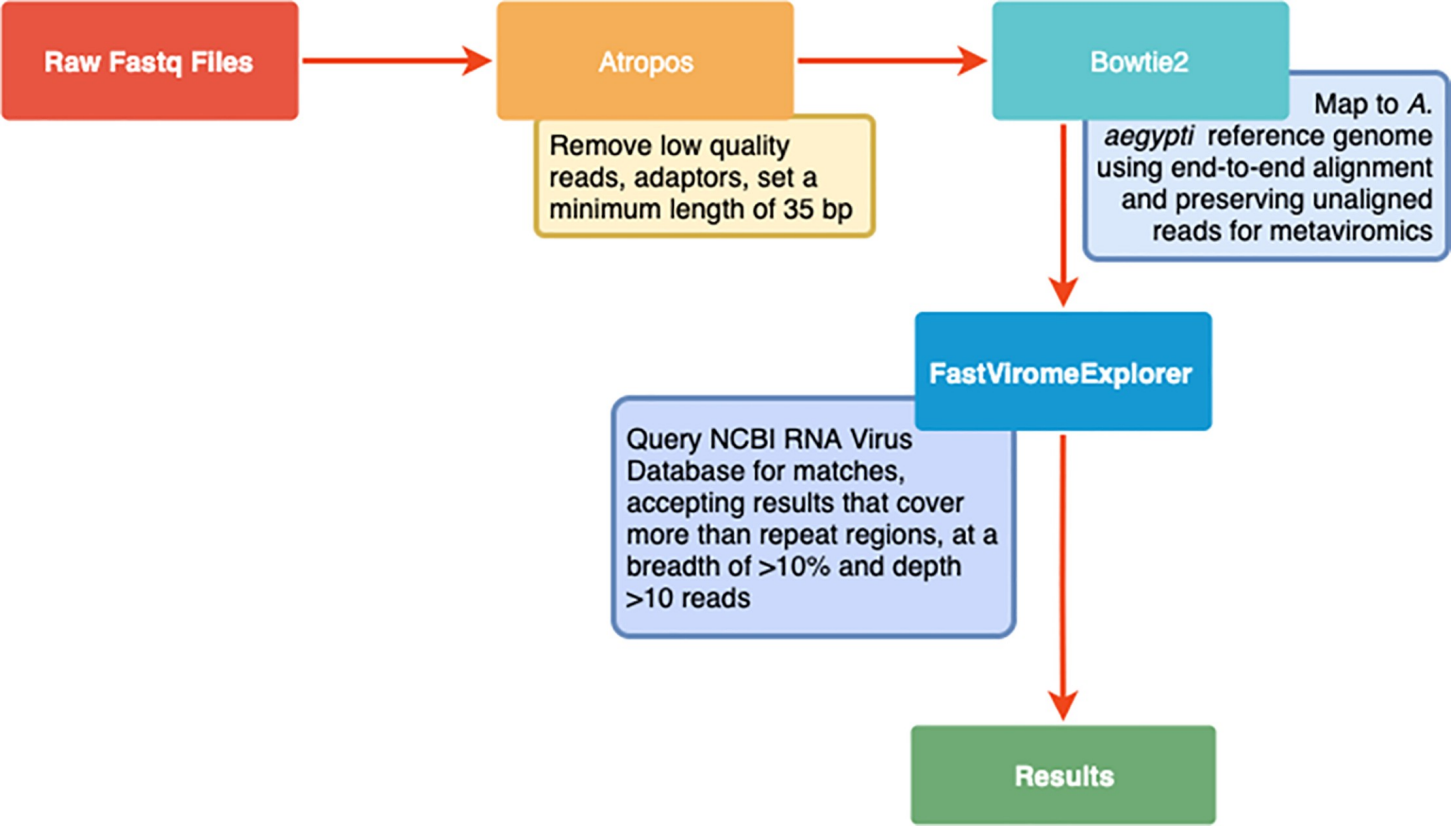
Metagenomic data analysis flow chart.

A metagenomic analysis flow chart can be found in Fig 2. Briefly, shotgun metagenomic sequencing was run using the Illumina HiSeq 1500 for deep sequencing. Raw fastq files were assessed for quality using Illumna FastQC version 1.0.0. Trimming and quality filtering of reads was performed using Atropos (https://omictools.com/atropos-tool), removing Illumina universal adaptors, reads with base calls below Q20, and a minimum length of 35 bp. Additional host read removal was performed bioinformatically using Bowtie2 (v. 2.3.4.3). Reads were mapped to the *A*. *aegypti* reference genome assembly AaegL5, available at https://www.vectorbase.org/organisms/aedes-aegypti, using end-to-end read alignment. Non-mosquito reads were analyzed using FastViromeExplorer [50] and the NCBI RNA Virus database as a reference, which contains a total of 23,085 contigs and is available at https://bench.cs.vt.edu/FastViromeExplorer/. FastViromeExplorer was run using the default parameters, which accounts for potential false positives by removing results that mapped to repeat regions of the genome, cover less than 10% of the genome, or had fewer than ten reads total. Estimated abundance is expressed as total read counts adjusted for segment size.

### RT-PCR verification of the metavirome results

About 200 ng of total RNA per pool (ten pools) or from individual mosquitoes was reverse transcribed using a High-Capacity cDNA Reverse Transcription Kit (ThermoFisher). RT-PCR was conducted on the resultant cDNA using previously published specific oligonucleotide primers (S1 Table). PCR amplicons of expected size were extracted from gels using the QIAquick Gel Extraction Kit (QIAgen, Hilden, Germany) following the manufacturer’s protocol. Amplicons were sent to the Molecular Cloning Lab, San Francisco, California (https://www.mclab.com/) for Sanger sequencing. Raw sequence data were manually edited using Chromas 2.6.5 software and then compared with the sequence database using the NIH’s Basic Local Alignment Search Tool (BLAST). Sequences of the Phasi Charoen-like virus (PCLV) were aligned with Clustal Omega (https://www.ebi.ac.uk/Tools/msa/clustalo/) to obtain a consensus sequence. The S-and M-segment consensus sequence were made with seven different sequences, and the L-segment with five different sequences obtained from different mosquito pools using the primers in S1 Table [51–55].

### Vertical transmission

Male *A*. *aegypti* mosquitoes, larva, and hatching eggs collected with oviposition traps as described in Jakob and Pratt [56] were pooled and used to determine potential vertical transmission. Larvae were confirmed as *A*. *aegypti* using pictorial keys for the identification of mosquitoes by Rueda [46]. RNA extraction, cDNA production and RT-PCR using PCLV primers were conducted as described above.

### Phylogenetic analysis of PCLV

Three phylogenetic trees were developed with the consensus sequences obtained by RT-PCR for the S, M, and L segments of PCLV. The multiple sequence alignments were produced with MAFFT version 7.0 [57] using the FFT-NS-2 progressive method. The trees were developed using neighbor-joining phylogeny based on the conserved regions and confidence testing by 1000 bootstrap replicates and visualized using Archaeopteryx.js. The phylogenetic analysis included several genera of the *Phenuiviridae* family (Order: *Bunyavirales*). A phylogenetic tree for the L segment of PCLV was also developed using the Bayesian Markov chain Monte Carlo method using MrBayes 3.2.6 (http://www.phylogeny.fr/one_task.cgi?task_type=mrbayes) (S1 Fig).

## Results and discussion

### RNA-seq results reveal a high prevalence of PCLV in *Aedes aegypti* in Grenada that is confirmed by RT-PCR

Assessment of Fastq files using FastQC showed an average read length of 125 bp. Total reads were 74,536,838 of which 74,273,783 remained after trimming. After depleting host reads using Bowtie2 (v.2.3.4.3), non-mosquito reads totaled 14,428,848 of which 171,792 could be classified as RNA virus. The predominant ISV in the metavirome was Phasi Charoen-like virus (PCLV) with 156,968 reads using the *A*. *aegypti* reference genome.

One of the limitations to metagenomic studies based on database analysis is the fact that sequence information for many viral families or genera is still limited and phylogenies based on single or short genome fragments can be misleading. This study used very stringent restrictions to establish high accuracy on the report of the virome using the *A*. *aegypti* assembly. PCLV was the most abundant virus and the only one that passed the restrictions (Table 1).

**Table 1:** *Aedes aegypti* metavirome in Grenada. Estimated abundances are presented as obtained using the reference genome for *A*. *aegypti*.

Virus identifier	Name	Family/Genus	Estimated Abundance
KU936056	Phasi Charoen-like virus strain Aag2-Bristol glycoprotein	*Phenuiviridae*/ Phasivirus	54,980.0
KM001085	Phasi Charoen-like virus RNA-dependent RNA polymerase		31,230.2
KR003786	Phasi Charoen-like virus isolate Rio segment L RNA-dependent RNA polymerase		24,713.8
KR003784	Phasi Charoen-like virus isolate Rio segment M glycoprotein		18,332.0
KM001086	Phasi Charoen-like virus glycoprotein precursor		16,483.0
KU936055	Phasi Charoen-like virus strain Aag2-Bristol nucleocapsid		5,020.5
KR003785	Phasi Charoen-like virusisolate Rio segment S nucleocapsid		3,790.9
KM001087	Phasi Charoen-like virus nucleocapsid		2,417.6

PCLV presence was confirmed with the same RNA used for library construction previous to pooling. A total of ten sub-pools, obtained from 30 mosquitoes each, were tested for PCLV by RT-PCR using the S-segment primers previously designed [51,52]. All PCLV amplicons were confirmed by Sanger sequencing and BLAST analysis, with PCLV as the best match with > 96% identity. Ten individual mosquitoes were also tested for PCLV using the S-segment and L-segment primers as well as an endogenous mosquito gene (see S1 Table). The presence of PCLV in all ten sub-pools (S2 Fig) and 70% of individual mosquitoes (S3 Fig) confirms the high prevalence of PCLV in *A*. *aegypti* mosquitoes from Grenada. The results also suggest that some *A*. *aegypti* are not infected with PCLV. We cannot rule out viral RNA degradation or poor primer sensitivity as explanations for why some mosquitoes were PCLV-negative by PCR. Regardless, future research should examine whether the PCLV infection status has an effect on the vector competence for arboviruses.

**Fig 3:**
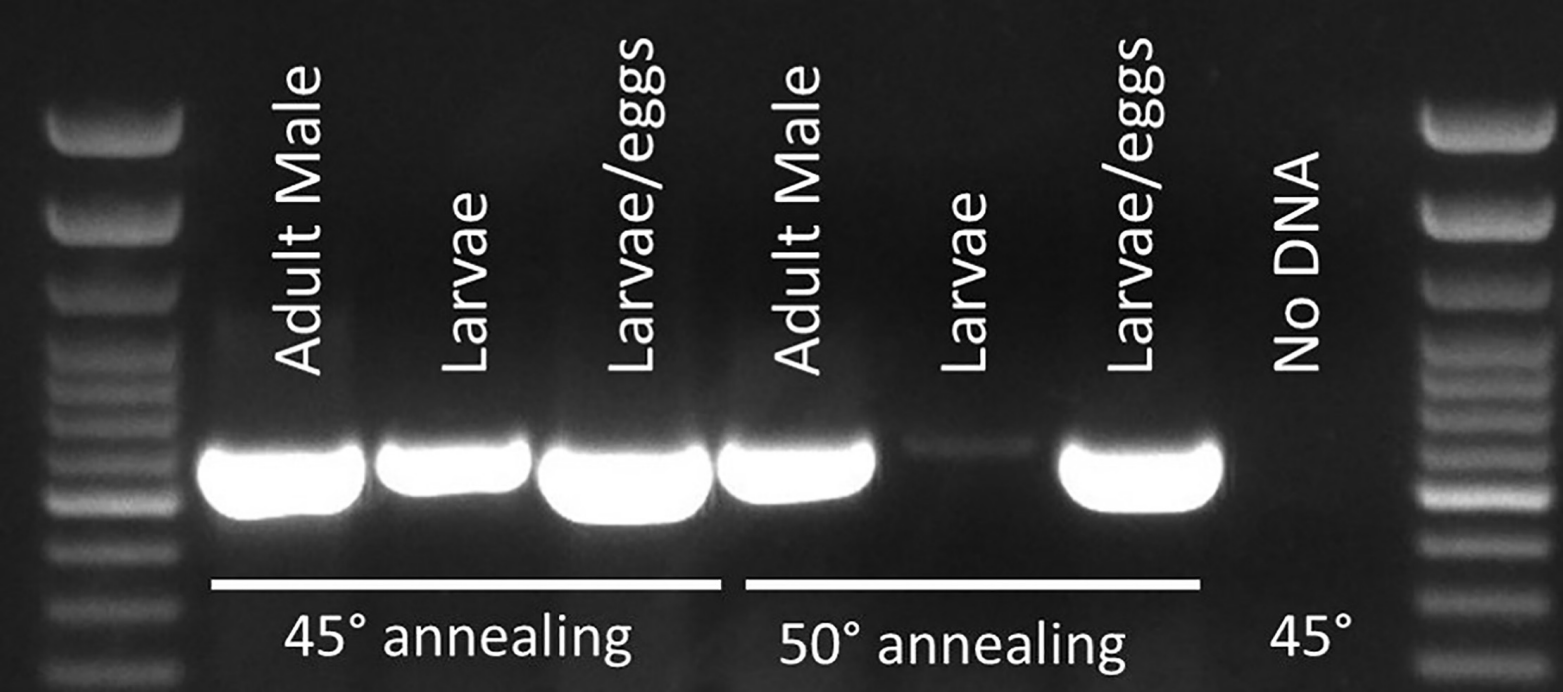
Detection of PCLV-Grenada in mosquito pools by RT-PCR in *Aedes aegypti* males, larvae, and hatching eggs (larvae/eggs).

PCLV was present not only in female mosquitoes, but also tested samples of males, hatching larvae (larva/eggs), and larvae of *A*. *aegypti* (Fig 3, S4 Fig), which suggests vertical transmission of the virus. Insect specific RNA viruses are often considered vertically transmitted due to their inability to replicate in mammalian cells and their presence in larvae and male adult forms, which do not feed on blood [42,58–60]. This points to the possibility that these viruses may have coexisted with their insect host for a long period of time [61–63] and have evolved with them [64].

Similar to our results, PCLV has been found to be the predominant ISV in *A*. *aegypti* in Australia, [25], Guadeloupe [23], South China [51], and Thailand [24]. *Aedes aegypti* originated in Africa [65]. There are two recognized subspecies of *A*. *aegypti* s.l.: 1) *A*. *aegypti formosus* restricted to Africa and 2) *A*. *aegypti aegypti*, found globally in tropical and subtropical regions typically in association with humans. Phylogenetic analyses of all mtDNA ND4 haplotypes reported to date for *A*. *aegypti* all support the hypothesis that *A*. *aegypti* populations from around the world consist of mosquitoes that arise from one of two matrilineages of African origin (reviewed in [66]). Moore et al. [66] further demonstrated this claim by sequencing ND4 haplotypes in 426 *A*. *aegypti* s.l. from Senegal (West Africa) and Kenya (East Africa) and finding all haplotypes found outside Africa matches two ancestral clades in Africa. When humans started living in groups and storing water, the larval breeding sites allowed for the *A*. *aegypti* mosquito to exploit the new niche [67] and for the females to adapt to the most available bloodmeal: human. This domesticated form of *A*. *aegypti* was likely introduced by the slave trade [65] from Africa into the Americas, from where it then spread to all other inhabited continents [68]. PCLV is highly prevalent in *A*. *aegypti* in all metavirome studies done so far (Australia, Asia, and America), and hence may have originated from the ancestral *Aedes* found in Africa and has evolved with the vector in different parts of the planet. ISVs and arboviruses show co-evolutionary relationship suggesting arboviruses could have been ISVs that through evolution acquired the ability to expand their host-range to vertebrates [42]. For example, in the order Bunyavirales, arthropod hosts have been constructed for all nodes of the bunyavirus tree [69], suggesting that arboviruses from this order may have evolved from ISVs. Similar examples can be found in other orders [42,70–73]. Because ISVs are RNA viruses with implied high mutation rates, together with the strong evolutionary pressure exerted on *A*. *aegypti* to live in areas densely populated with humans and other peridomestic animals, the opportunity for host expansion is a possibility and has been previously suggested for genera like the hantaviruses [74].

The impact of PCLV on the *A*. *aegypti* lifecycle and vector competency requires further study. It has been reported that the presence of PCLV in the mosquito may inhibit the replication of some arboviruses. For example, dual infection with both PCLV and Cell-fusing agent virus in *A*. *albopictus* cell line As23 results in replication inhibition of two flaviviruses (ZIKV and DENV) and the bunyavirus La Crosse virus [75]. In contrast, a study using a pre-existing persistent PCLV infection had no major impact on the replication on DENV, ZIKV, the alphavirus Sindbis virus, or the rhabdovirus vesicular stomatitis virus [76]. The discrepancy found in studies using cell lines may be explained by the previous infection status of the cells used in the different studies, which points to the need to do studies in live mosquitoes. There have been two studies looking at the effects of ISVs on vector competence for arboviruses using live mosquitoes, both associated with WNV in *C*. *quinquefasciatus* [34,77] but none on PCLV.

### RNA-seq and RT-PCR show absence of alphaviruses and flaviviruses in the sample pools

The mosquito metagenomic analysis did not show the presence of any of the most common endemic or recently introduced arboviruses in Grenada. To confirm the absence of alphaviruses (Family: *Togaviridae*) including chikungunya virus and flaviviruses like ZIKV and DENV in the sample pools, nested RT-PCR was run using genus-level primers as indicated in S1 Table. For the alphaviruses, previously used primers targeting the highly conserved nsP4 coding region of alphavirus RNA was chosen as described previously [54]. No amplicons for alphaviruses were detected for any of the mosquito pools. PCR amplicons of the expected size obtained with the pan-flavivirus primers were sequenced showing the bands belong to ISV-flaviviruses. The detection of low abundance viruses is likely limited in metagenomic analysis by the stringent methods described above.

### Phylogenetic tree shows the relationship between PCLV-Grenada, reported PCLVs, and other *Phenuiviridae* viruses

**Fig 4:**
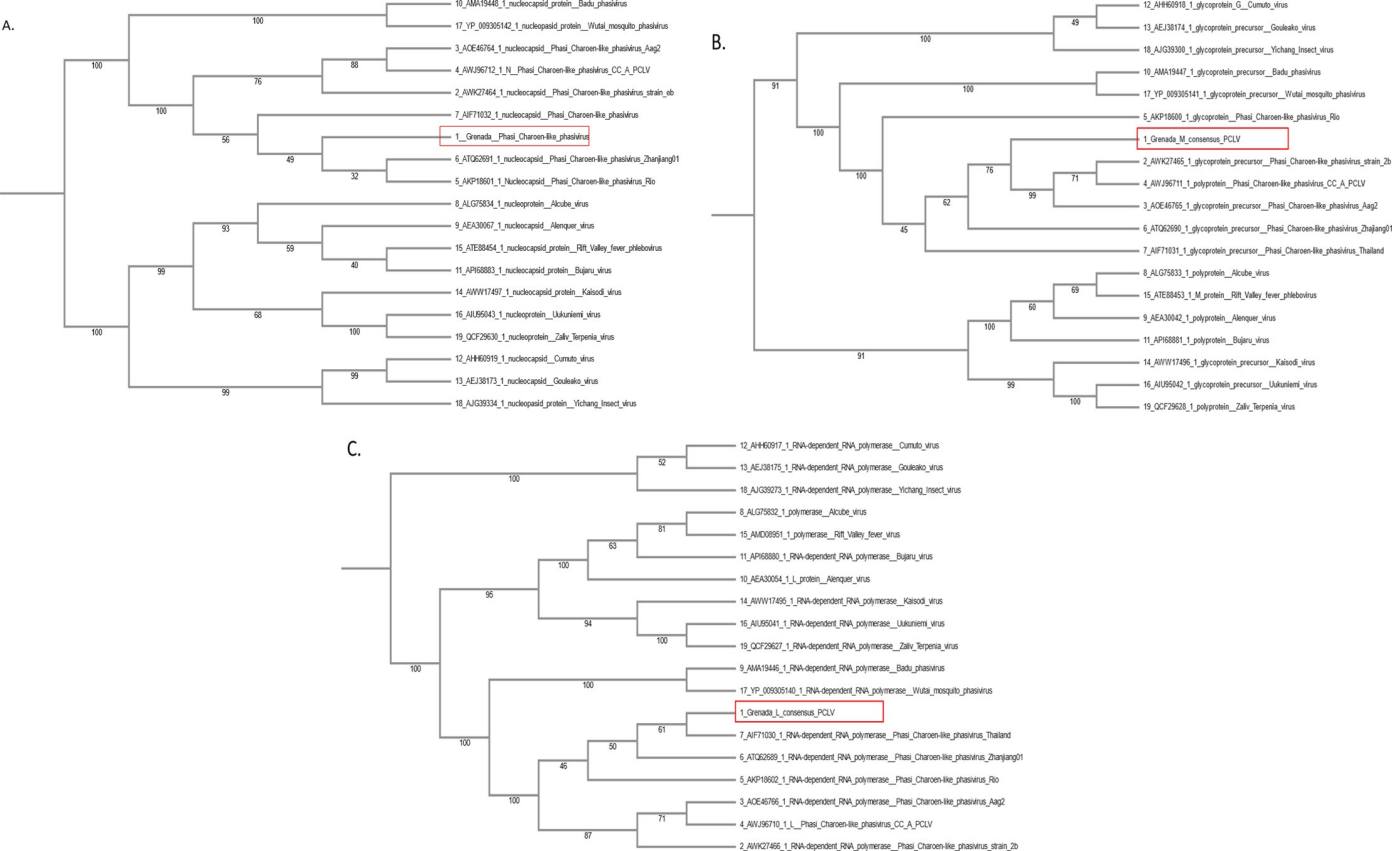
Phylogenetic trees showing the relationship between PCLV-Grenada and other *Phenuviridae*. Trees were developed using MAFFT version 7.0’s FFT-NS-2 progressive method as the strategy. The trees were developed using NJ phylogeny based on the conserved regions and confidence testing by 1000 bootstrap replicates. A. S segment, B. M segment, and C. L segment.

Phylogenetic trees of all three segments (S, M, and L) of PCLV were constructed using published data of taxa from the *Phenuiviridae* Family in GenBank against the consensus of six independent sequences of PCLV found in Grenada. PCLV in this study shows a closer association to the *Phasivirus* genus, in all phylogenetic trees, than to the *Goukovirus* or *Phlebovirus* genera in this family (Fig 4 and S4 Fig) according to the latest report of ICTV released in 2018 (https://talk.ictvonline.org/). These findings are in disagreement with previous reports [78] where PCLV was grouped with the newly proposed genus *Goukovirus* because it clustered together with Badu virus and Gouleaku virus, but in agreement with results from similar studies in South China [51]. The consensus sequence of The PCLV obtained in Grenada had a 96–97% similarity to all other published PCLV sequences in GenBank as determined by BLAST. The sequences for the PCLV Grenada isolate have been deposited in GenBank under the accession numbers MN109951 (for the S segment/nucleocapsid coding region), MN109952 (for the M segment/glycoprotein coding region), and MN109953 (for the L segment/RNA dependent RNA polymerase coding region).

## Conclusions

In summary, we have shown here that in the Caribbean, *Aedes aegyp*ti most abundant virus is PCLV, as has been reported previously in Guadeloupe and also in other continents. The similarities found in *A*. *aegypti* virome worldwide, and particularly the presence of PCLV has many implications, including the likelihood of a common ancestry. Future studies in Africa will be needed to link PCLV to ancestral origins. Due to the widespread presence of this ISVs in *A*. *aegypti*, the impact of PCLV in this mosquito’s biology and vector competency needs to be addressed in wild populations. This study used pooling of samples and stringent restrictions while analyzing the virome of *Aedes aegypti* which may have affected the report on low abundance viruses.

## Supporting information

S1 Fig Phylogenetic trees for the L segment of PCLV using the Bayesian Markov chain Monte Carlo method. (PDF)

S2 Fig Detection of PCLV-Grenada in 10 mosquito sub-pools by RT-PCR. Controls include: a mosquito pool previously determined to be PCLV-positive, extraction control (negative), and no DNA control (negative).
(TIFF)

S3 Fig Detection of PCLV-Grenada in 10 individual mosquitoes by RT-PCR. A. PCLV S-segment. B. PCLV L-segment. C. Mosquito endogenous gene (AAEL004181). Controls include: a mosquito pool previously determined to be PCLV-positive, extraction control (negative), and no DNA control (negative).
(TIF)

S4 Fig Uncropped Fig 3. (TIF)

S5 Fig Uncropped S3 Fig. A. Gel electrophoresis for PCLV S-segment primer PCR. B. Gel electrophoresis for PCLV L-segment primer PCR (top) and mosquito endogenous gene (bottom).
(TIF)

S1 Table Primers used in this study. (PDF)
