# Supplementary material for: High prevalence of Phasi Charoen-like virus from wild-caught Aedes aegypti in Grenada, W.I. as revealed by metagenomic analysis
Source: PLoS One. 2020 Jan 31;15(1):e0227998. doi: 10.1371/journal.pone.0227998 (PMC6993974; doi:10.1371/journal.pone.0227998)
Supplement: S1 Fig — (PDF) [file pone.0227998.s001.pdf]

Clade credibility values:

```
/------------------------------------------------------ AJG39273.1 (1)
|
|                                    /------- AHH60917.1 (2)
|---------------------87--------------------+
|                                    \------- AEJ38175.1 (3)
|
|                         /------------------ AKP18602.1 (4)
|                         |
|                         |          /------ AOE46766.1 (5)
|                     /--64--+    /---53--+
|                     |    |    |     \------- AWJ96710.1 (7)
|                     |    \---80--+
|                     |         \------------ AWK27466.1 (6)
+                /---55--+
|                |     |----------------------- AIF71030.1 (8)
|                |     |
|          /---94--+    \----------------------- ATQ62689.1 (10)
|          |     |
|          |     \----------------------------- Grenada_L (9)
|     /---96--+
|     |     |                       /------- AMA19446.1 (11)
```

Goukovirus

Phasivirus

Legend: *Taxon names association table*

Output Taxon Name	Original (Long) Taxon Name
AEA30054.1_L_protein_Alenquer_virus	AEA30054.1_L_protein_[Alenquer_virus]
AEJ38175.1_RNA-dependent_RNA_polymerase_Gouleako_virus	AEJ38175.1_RNA-dependent_RNA_polymerase_[Gouleako_virus]
AHH60917.1_RNA-dependent_RNA_polymerase_Cumuto_virus	AHH60917.1_RNA-dependent_RNA_polymerase_[Cumuto_virus]
AIF71030.1_RNA-dependent_RNA_polymerase_Phasi_Charoen-like_phasi	AIF71030.1_RNA-dependent_RNA_polymerase_[Phasi_Charoen-like_phasivirus_Thailand
AIU95041.1_RNA-dependent_RNA_polymerase_Uukuniemi_virus	AIU95041.1_RNA-dependent_RNA_polymerase_[Uukuniemi_virus]
AJG39273.1_RNA-dependent_RNA_polymerase_Yichang_Insect_virus	AJG39273.1_RNA-dependent_RNA_polymerase_[Yichang_Insect_virus]
AKP18602.1_RNA-dependent_RNA_polymerase_Phasi_Charoen-like_phasi	AKP18602.1_RNA-dependent_RNA_polymerase_[Phasi_Charoen-like_phasivirus_Rio]
ALG75832.1_polymerase_Alcube_virus	ALG75832.1_polymerase_[Alcube_virus]
AMA19446.1_RNA-dependent_RNA_polymerase_Badu_phasivirus	AMA19446.1_RNA-dependent_RNA_polymerase_[Badu_phasivirus]
AMD08951.1_polymerase_Rift_Valley_fever_virus	AMD08951.1_polymerase_[Rift_Valley_fever_virus]
AOE46766.1_RNA-dependent_RNA_polymerase_Phasi_Charoen-like_phasi	AOE46766.1_RNA-dependent_RNA_polymerase_[Phasi_Charoen-like_phasivirus_Aag2]
API68880.1_RNA-dependent_RNA_polymerase_Bujaru_virus	API68880.1_RNA-dependent_RNA_polymerase_[Bujaru_virus]
ATQ62689.1_RNA-dependent_RNA_polymerase_Phasi_Charoen-like_phasi	ATQ62689.1_RNA-dependent_RNA_polymerase_[Phasi_Charoen-like_phasivirus_Zhanjian
AWJ96710.1_L_Phasi_Charoen-like_phasivirus_CC_A_PCLV	AWJ96710.1_L_[Phasi_Charoen-like_phasivirus_CC_A_PCLV]
AWK27466.1_RNA-dependent_RNA_polymerase_Phasi_Charoen-like_phasi	AWK27466.1_RNA-dependent_RNA_polymerase_[Phasi_Charoen-like_phasivirus_strain_2
AWW17495.1_RNA-dependent_RNA_polymerase_Kaisodi_virus	AWW17495.1_RNA-dependent_RNA_polymerase_[Kaisodi_virus]
Grenada_L_consensus_PCLV	Grenada_L_consensus_PCLV
QCF29627.1_RNA-dependent_RNA_polymerase_Zaliv_Terpenia_virus	QCF29627.1_RNA-dependent_RNA_polymerase_[Zaliv_Terpenia_virus]
YP_009305140.1_RNA-dependent_RNA_polymerase_Wutai_mosquito_phasi	YP_009305140.1_RNA-dependent_RNA_polymerase_[Wutai_mosquito_phasivirus]
