# Supplementary figures and images for: High prevalence of Phasi Charoen-like virus from wild-caught Aedes aegypti in Grenada, W.I. as revealed by metagenomic analysis
Source: PLoS One. 2020 Jan 31;15(1):e0227998. doi: 10.1371/journal.pone.0227998 (PMC6993974; doi:10.1371/journal.pone.0227998)

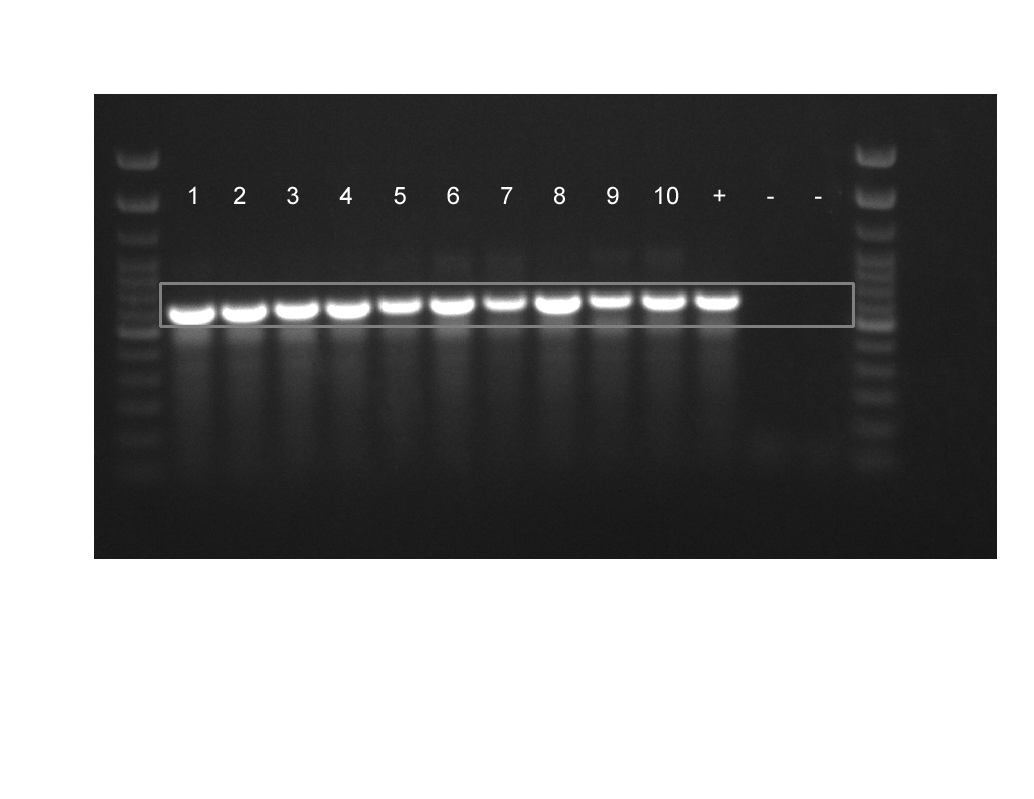

Supplement: S2 Fig — Controls include: a mosquito pool previously determined to be PCLV-positive, extraction control (negative), and no DNA control (negative). (TIFF) [file pone.0227998.s002.tiff]

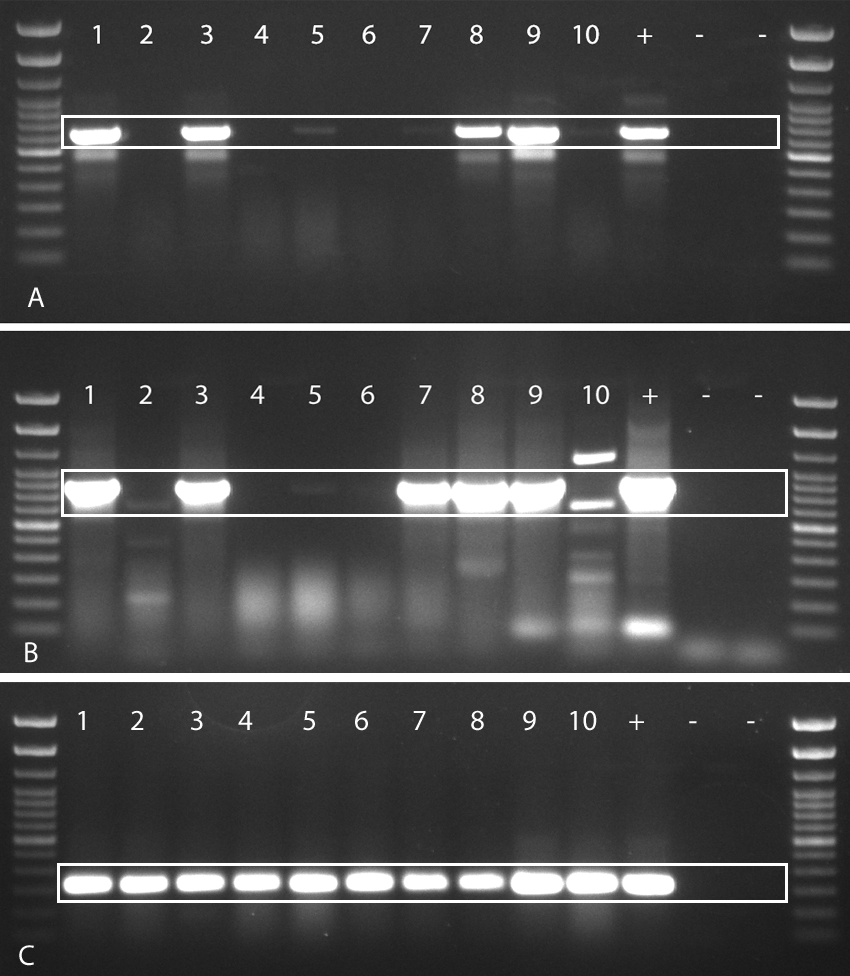

Supplement: S3 Fig — A. PCLV S-segment. B. PCLV L-segment. C. Mosquito endogenous gene (AAEL004181). Controls include: a mosquito pool previously determined to be PCLV-positive, extraction control (negative), and no DNA control (negative). (TIF) [file pone.0227998.s003.tif]

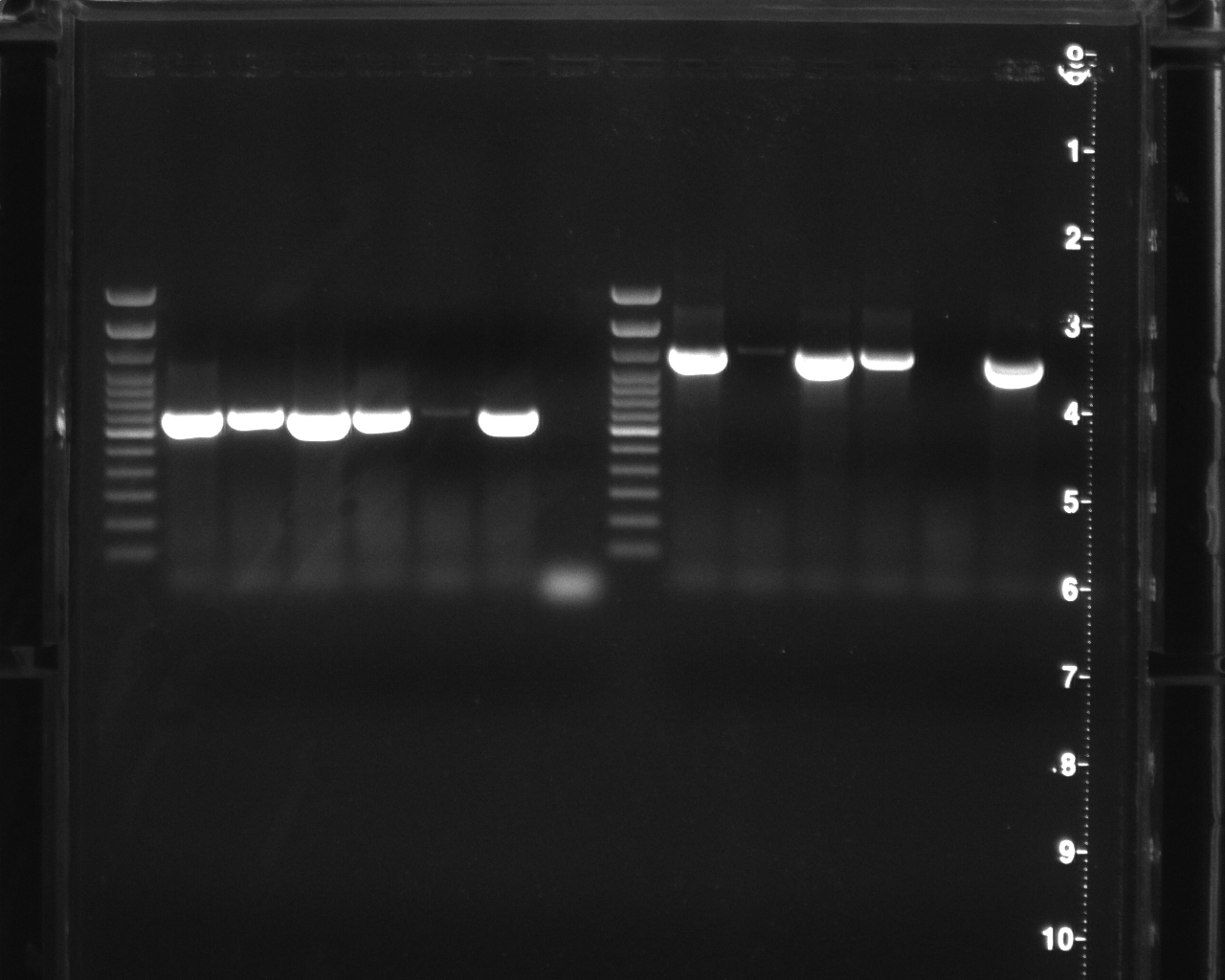

Supplement: S4 Fig — (TIF) [file pone.0227998.s004.tif]

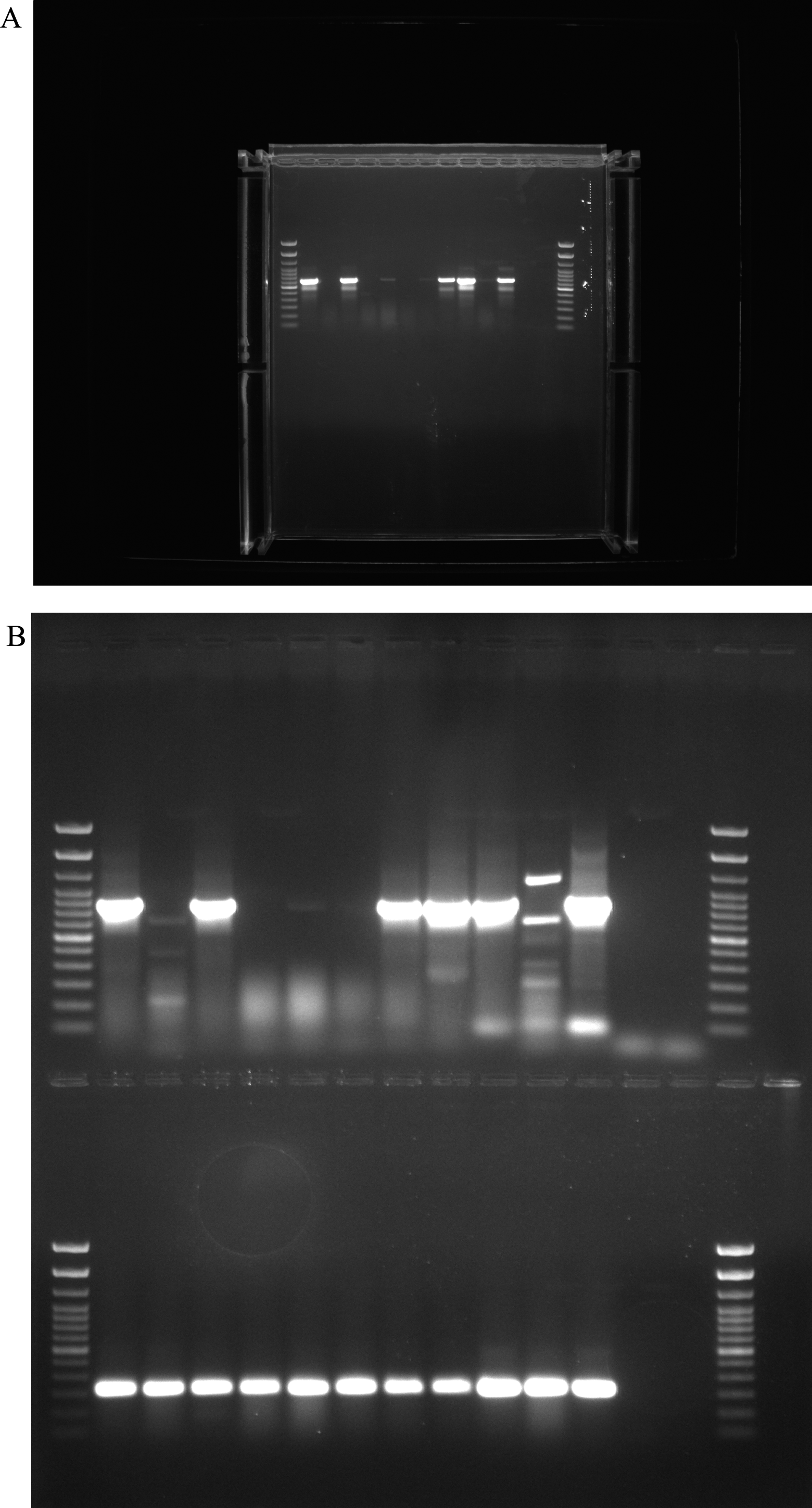

Supplement: S5 Fig — A. Gel electrophoresis for PCLV S-segment primer PCR. B. Gel electrophoresis for PCLV L-segment primer PCR (top) and mosquito endogenous gene (bottom). (TIF) [file pone.0227998.s005.tif]
