# Supplementary material for: High prevalence of Phasi Charoen-like virus from wild-caught Aedes aegypti in Grenada, W.I. as revealed by metagenomic analysis
Source: PLoS One. 2020 Jan 31;15(1):e0227998. doi: 10.1371/journal.pone.0227998 (PMC6993974; doi:10.1371/journal.pone.0227998)
Supplement: S1 Table — (PDF) [file pone.0227998.s006.pdf]

S1 Table. Primers used in this study

Viruses	Primers (5' to 3')	Target	Reference
PCLV	L segment-F:<br>AGACAGCACAAGCAAATAAAGCAAG<br>L segment-R:<br>AAACATGCATTGTAAGGTTTTGTCG<br>M segment-F:<br>AAAAGTAGGAATTGATGCTGTTGC<br>M segment-R:<br>CTTTGAGCACTTTTGTCTAATGGC	M and L segments	[51]
	PCLV-N-F:<br>CAGTTAAAGCATTTAATCGTATGATAA<br>S segment-R:<br>TGGAAAATAAAAACAATAAAGCAATAC	S segment	[52] forward primer<br>[51] reverse primer
Pan-Flavivirus	First PCR:<br>MAMD - AACATGATGGGRAARAGRGARAA<br>cFD2 - GTGTCCCAGCCGGCGGTGTCATCAGC<br>Second PCR:<br>FS 778 - AARGGHAGYMCDGCHATHTGGT<br>cFD2 - GTGTCCCAGCCGGCGGTGTCATCAGC	NS5	[53]
Pan-Alphavirus	First PCR:<br>TTTAAGTTTGGTGCGATGATGAAGTC-F<br>GCATCTATGATATTGACTTCCATGTT-R<br>Second PCR:<br>GGTGCGATGATGAAGTCTGGGATGT-F<br>CTATGATATTGACTTCCATGTTCATCCA-R1<br>CTATGATATTGACTTCCATGTTCAGCCA-R2	nsP4 coding region	[54]
Endogenous mosquito gene	ACAACCAGTGGAATCCTTCG-F<br>GTTCTCATTTGCGACCCAAT-R	AAEL004181	[55]
